# Are Adolescent Body Image Concerns Associated with Health-Compromising Physical Activity Behaviours?

**DOI:** 10.3390/ijerph16071225

**Published:** 2019-04-05

**Authors:** Rasa Jankauskiene, Migle Baceviciene, Simona Pajaujiene, Dana Badau

**Affiliations:** 1 Institute of Sport Science and Innovation, Lithuanian Sports University, Sporto 6, 44221 Kaunas, Lithuania; rasa.jankauskiene@lsu.lt; 2 Department of Health, Physical and Social Education, Lithuanian Sports University, Sporto 6, 44221 Kaunas, Lithuania; migle.baceviciene@lsu.lt; 3 Department of Coaching Science, Lithuanian Sports University, Sporto 6, 44221 Kaunas, Lithuania; simona.pajaujiene@lsu.lt; 4 Department of Human Movement Sciences, University of Medicine, Pharmacy, Sciences and Technology of Targu Mures, 540139 Targu Mures, Romania

**Keywords:** adolescence, body image, excessive exercise, health-compromising behaviours, dieting

## Abstract

The present study aimed to assess the-prevalence of health-compromising eating and physical activity behaviours, and to test their associations with physical activity, internalisation of sociocultural attitudes towards appearance, and body image in a sample of adolescents of both genders. A total sample of 736 adolescents (437 or 59.4% were girls) participated in the study. The participants ranged in age from 16 to 19 years (x = 17.2, SD = 0.6). The sample completed a questionnaire measuring body mass index, the risk of eating disorders, body image, internalisation of sociocultural ideals of appearance, health-compromising eating behaviours (HCEB), and health-compromising weight control related to physical activity behaviours (HCPAB). Logistic regressions were used to assess the associations between the study variables and predictors of HCEB and HCPAB. The results of the study showed a relatively high prevalence of HCEB with a significantly higher prevalence in girls and participants with a higher BMI. The study also demonstrated that the prevalence of adolescent HCPAB was higher than HCEB. The internalisation of sociocultural attitudes towards appearance and body image concerns were higher in the HCEB and HCPAB groups. Female gender (OR = 1.88; 95% PI = 1.10–3.18), HCPAB (OR = 1.19; 95% PI = 1.10–1.28), a preoccupation with being overweight (OR = 3.43; 95% PI = 2.52–4.66), and body weight evaluation as too high (OR = 2.40; 95% PI = 1.57–3.68) were significant predictors of HCEB. More frequent physical activity (OR = 3.02; 95% PI = 1.76–5.17), HCEB (OR = 1.22; 95% PI = 1.11–1.32), and perceived pressures to conform to popular beauty ideals (OR = 1.51; 95% PI = 1.12–2.03) predicted higher HCPAB. HCPAB is an important variable associated with adolescents’ body image, physical activity, and weight control. The results of the present study are important for health promotion and education programs addressing adolescents’ healthy lifestyle, weight control, and body image concerns.

## 1. Introduction

Physical activity (PA) plays an important role in the health of adolescents [1]. However, a significant proportion of adolescents do not meet the recommendations for PA. In Lithuania, only 23% of 15 year old boys and 12% of girls meet WHO physical activity recommendations [2] and there has been a decline in physical fitness in recent decades [3]. Compared to other countries, Lithuanians have one of the lowest incidences of overweight and obesity in 7 to 17 year old children and adolescents in Europe [4], but studies show that the body mass index (BMI) of Lithuanian children is increasing [4] and a significant number of adolescents have body image concerns [5].

Adolescence is a time of rapid emotional, social, and physical change. Those changes and the internalisation of sociocultural ideals play an important role in the development of adolescent body dissatisfaction. Studies have demonstrated that media use, gender norms, and stereotypes about appearance were associated with increased body image concerns and body dissatisfaction [6,7,8]. Adolescents’ body dissatisfaction is associated with a higher BMI, dieting, lower self-esteem, poorer perceived health, more time spent on computers, prevalence of unhealthy weight loss strategies, increased risk of eating disorders, sadness, suicidal ideation, lower physical activity, external physical activity motivation, and dysfunctional exercise [7,9,10,11,12,13,14,15,16,17]. The prevalence of body dissatisfaction in adolescent girls is higher than in boys [13] and overweight or obese adolescents demonstrate higher body dissatisfaction than adolescents of a normal body weight [18,19]. Studies show that a progressive increase in body dissatisfaction from adolescence to young adulthood is related to the development of overweight and obesity and to eating disorders in later life [20,21].

A plethora of studies in samples of Western adolescents suggest that adolescent body image concerns are associated with the prevalence of health-compromising eating-related behaviours (HCEB), such as restricted food intake, the use of laxatives and diuretics, purging, the use of diet pills, and induced vomiting [22,23,24]. Studies show that HCEB are related to being overweight and obesity, body weight overestimation, higher body dissatisfaction, lower self-esteem, perfectionism, higher depression, delinquency, suicide ideation, and reported suicide attempts [11,18,23,24,25,26,27,28]. Conversely, Lampard et al. (2016) showed that exclusively healthy weight control behaviours were more prevalent in adolescent girls who were not overweight compared to overweight and obese girls [29]. The majority of studies analysing health-compromising behaviours have focused on eating and eating- related compensatory behaviours [15,22,24,29]. Studies, however, show that a significant number of adolescents attempting to lose weight also use physical activity or exercising strategies as well [29,30,31]. In their representative study, Lee and Lee [32,33] found that among adolescents attempting to control their weight, the most common weight control behaviour was engaging in regular exercise.

Increasing physical activity and exercise is healthy adolescents’ weight control behaviour [29,30]. However, to date, it is unclear how adolescents use physical activity when they decide to engage in weight control. Studies show that in some cases, exercising might become dysfunctional if quantitative and qualitative principles of exercise for health are ignored [34]. Current guidelines do not define excessive exercising, yet excessive physical activity manifests in psychological and physiological symptoms and is defined as exercise addiction [35]. Studies show that, like disordered eating behaviours, dysfunctional exercising compromises health and affects physical and psychological health [36,37]. However, examples from practice and some initial studies show that physical activity for weight control might be associated with over-exercising in terms of intensity and time, wearing impermeable clothes that allow greater sweating, and not drinking fluids after exercise in order to decrease body weight [37]. This behaviour may be seriously health damaging. The motivation for this behaviour might be associated with poor physical literacy in the physical activity-related weight control knowledge domain. The term “physical literacy” is relatively novel and means that individuals possess the appropriate motivation, confidence, knowledge, skills, and fitness necessary to enjoy a physically active lifestyle and are committed to healthy habitual movement behaviours [38]. Thus, it is important to understand if and how adolescents use health compromising physical activity behaviours for weight control and to explore its prevalence, predicting factors, and correlates.

Therefore, the aim of the present study was to assess the prevalence of health-compromising eating and physical activity-related behaviours in a sample of adolescents, and to test its associations with physical activity, the internalisation of sociocultural attitudes about appearance, and body image concerns. We expected that health-compromising eating and physical activity behaviours would be more prevalent among girls and adolescents with a higher BMI. We also assumed that adolescents who engage in health-compromising eating behaviours will demonstrate a higher prevalence of health-compromising physical activity behaviours. We expected that a higher internalisation of sociocultural attitudes towards appearance and body image concerns would be associated with higher health-compromising eating and physical activity behaviours, controlled for by gender and BMI. Finally, we expected that more physical activity would be associated with a lower prevalence of health-compromising eating and exercising behaviours.

## 2. Materials and Methods

### 2.1. Research Design

This cross-sectional survey included adolescents from 16 secondary schools in Kaunas (the second largest city in Lithuania). Schools were randomly selected from the institutional registry list of the Ministry of Education and Science. The study was approved by the Kaunas municipality Education Department (No. 35-2-570) and Ethical Board of Institute of Social Sciences of Lithuanian Academy of Physical Education (protocol No. 3). Written informed consent was obtained from all study participants.

Questions were asked during lessons in schools by a group of trained researchers. The respondents provided their answers by completing questionnaires consisting of a battery of self-report instruments designed to measure the study variables. It took approximately 45 minutes to complete the questionnaire. Eight hundred and fifty-six questionnaires were completed, and 736 questionnaires were used for this study. The excluding criteria was that not all items of the questionnaires were filled in by the students.

### 2.2. Participants

In total, 736 adolescents (437 or 59.4% were girls) participated in the study. The participants were in the 11th grade of public high school in Kaunas (the second largest city of Lithuania). The participants ranged in age from 16 to 19 years (x = 17.2, SD = 0.6).

### 2.3. Measures

Demographics. Participants were asked to indicate their age and date of birth.

Body mass index (BMI) was assessed using self-reported height and weight from which BMI was calculated (kg/m^2^).

The mean body mass index (BMI) of the students was calculated as the individual’s weight divided by their height squared. As recommended by the International Obesity Task Force (IOTF) cut-offs, the sample was classified into four body mass categories according to percentiles: Below the 5th percentile was thin, between the 5th and 84th was normal weight, between the 85th and 94th was overweight, and the 95th percentile and above was obese [39]. In the further analyses, the overweight and obesity categories were combined. The results showed that 12.0% of boys and 4.8% of girls were classed as either overweight or obese. Research suggests that self-reported anthropometric measurements in young adults can be used to calculate BMI for weight classification purposes [40].

Body weight discrepancy (BWD) was measured as the difference between self-reported body weight and perceived ideal body weight. This measure shows the dissatisfaction with current body weight. The higher the discrepancy, the greater the dissatisfaction with individuals’ current body weight.

Health-compromising eating behaviours (HCEB) included specific behaviours that were not professionally recommended for weight reduction or are health damaging [14,22,23]. Those behaviours were assessed through the question “Have you ever done any of the following things in order to lose weight or keep from gaining weight in your life?”. The following behaviours were included: (1) Skip meals; (2) fast; (3) smoke more cigarettes; (4) eat very little food (less than 800 kcal); (5) use diuretics; (6) eat only one product or liquid while dieting; (7) take diet pills to suppress appetite; (8) make yourself vomit; (9) use laxatives. Each method was rated on a Likert scale (from 1 = “not all true for me” to 5 = “very true for me”). Reporting the use of any of these weight control behaviours was categorised as engaging in health-compromising eating-related behaviours, with the Cronbach alpha as 0.84 and the test-retest reliability as ICC = 0.87.

Health-compromising physical activity behaviours (HCPAB) included specific physical activity and exercise-related behaviours that are not recommended by exercise and health professionals and might be health damaging. Those behaviours were assessed through the question “Have you ever done any of the following things in order to lose weight or keep from gaining weight in your life?”. The following behaviours were included: (1) Exercise as long as I can (more than 2 h); (2) exercise several times per day; (3) avoid drinking fluids while exercising; (4) wear warm or impermeable clothes to sweat more; (5) exercise at maximum intensity; (6) participate in several workouts at once. Each method was rated on a Likert scale (from 1 = “not at all true for me” to 5 = “very true for me”). Reporting the use of any of these weight control behaviours was categorised as engaging in health-compromising physical activity-related behaviours, with the Cronbach alpha as 0.77 and a test-retest reliability of ICC = 0.89.

Physical Activity (PA). We examined how frequently physical activity had been performed for 60 minutes or more per day in the last seven days (this item is used in the WHO questionnaires conducted by the international study of health behaviour in school-aged children). Adolescents reporting exercising 0 to 1 times weekly during their leisure time were classified as physically inactive, 2 to 3 times as insufficiently active, and 4 times or more as active. 

The Lithuanian version of the short version of the Multidimensional Body and Self Regulations Questionnaire-AS (MBSRQ-AS) [41] was used. This instrument evaluates the appearance-related components of the body image construct [41]. It consists of 7 items on the appearance evaluation scale, which measures feelings of physical attractiveness and satisfaction with one’s looks, with higher scores indicating a higher appearance evaluation. The 12-item appearance orientation scale assesses the extent of investment in one’s appearance and higher scores indicate a higher appearance orientation. The overweight preoccupation scale assesses fat anxiety, weight vigilance, dieting, and eating restraint and consists of 4 items. Higher scores on this scale show a greater preoccupation with being overweight. The body areas satisfaction scale (9 item) assesses satisfaction or dissatisfaction with specific areas of the body on a five point scale (complete satisfaction to complete dissatisfaction). The self-classified weight scale consists of two items and reflects on how one perceives and labels one’s weight (ranging from very underweight to very overweight). A higher score shows higher beliefs that body weight is too high. The Lithuanian version of MBSRQ-AS has been established in the literature [42]. The scale was obtained from the official site with the official permission of the author. Internal consistency of the scales (appearance evaluation, appearance orientation, body areas satisfaction, overweight preoccupation, self-classified weight) was, respectively, 0.73; 0.73; 0.87; 0.76, and 0.76.

The Lithuanian version of The Rosenberg’s Self-Esteem Scale [43], is the most widely used measure of global self-esteem and has been determined to be valid and reliable among students. The scale consists of 10 items rated on a four-point Likert scale (from 1 = “not at all true for me” to 4 = “very true for me”), yielding scores from 10 to 40. Higher scores reflect a greater level of self-esteem. For the present study, the internal consistency of the scale was good (Cronbach α = 0.76).

The Lithuanian version of The Sociocultural Attitudes towards Appearance Questionnaire-3 (SATAQ-3) [44] is a measure of one’s endorsement of societal appearance ideals. The original SATAQ-3 has 30 items and four subscales: Internalisation-general (9 items), internalisation-athlete (5 items), pressures (7 items), and information (9 items). Each of these is composed of items that are rated on a 5-point Likert-type scale (1 = “Definitely disagree”, 5 = “Definitely agree”). The higher the score, the greater the acceptance or internalisation of the prevailing sociocultural standards for appearance. Research supports the use of the questionnaire with Lithuanian adolescents [45]. For the current sample, the Cronbach’s alphas were 0.71 for internalisation-general, 0.68 for internalisation-athlete, 0.77 for pressure, and 0.68 for information.

### 2.4. Statistical Analysis

Firstly, descriptive statistics and distribution normality testing of continuous variables were performed. Secondly, preliminary analyses (χ^2^, Mann-Whitney, independent sample t test, Spearman correlations,) were conducted to examine associations between variables. Finally, binary multiple logistic regression analysis models were employed to estimate the effect of different factors on HCEB and HCPAB (dichotomised as 0—no, 1—yes). Adolescents who answered, “not all true to me” or “not true to me” were classified as free from HCBAB and HCEB while the rest were classified as using these strategies for their weight control. HCEB (when predicting HCPAB) and HCPAB (when predicting HCEB) were entered into the models as continuous variables with higher scores indicating more frequent use of particular strategies. The Hosmer-Lemeshow goodness of fit test was conducted to test the data fit for logistic regression. All variables included in the logistic regression model were entered in a single step (enter method). For this study, the significance threshold considered relevant was *p* < 0.05, 95% confidence interval (CI). The reliability or the internal consistency of the questionnaires was calculated with index Cronbach’s alpha statistical (α). Statistical analyses were conducted using IBM SPSS Statistics 25 (IBM Corp., Armonk, NY, USA).

## 3. Results

Sample characteristics are presented in Table 1. The majority of the sample was of a normal weight. Underweight was more prevalent among girls, whereas overweight was more prevalent among boys. Boys’ physical activity was significantly higher than girls’ physical activity (*p* = 0.002). Girls demonstrated a greater desire to decrease body weight (*p* < 0.0001), higher appearance orientation (*p* < 0.0001), lower body satisfaction (*p* < 0.0001), and higher overweight preoccupation (*p* < 0.0001). There were also significant differences in all SATAQ-3 scales (with the exception of sports internalisation), with girls demonstrating significantly higher scores on the SATAQ-3 subscales (*p* < 0.0001).

We explored the prevalence of HCEB and HCPAB in boys and girls (Table 2). The results demonstrated a significantly higher prevalence of HCEB in girls, with exceptions for increased cigarette smoking and different medications used in order to reduce body mass. HCPA behaviour did not differ significantly between boys and girls.

Next, we explored the associations between HCEB and the study variables (Table 3). Respondents with HCEB reported a greater desire to lose body weight (*p* < 0.0001), lower self-esteem (*p* = 0.015), and physical activity (*p* = 0.01). HCEB was associated with a lower appearance evaluation (*p* < 0.0001), higher appearance orientation (*p* < 0.0001), lower body satisfaction (*p* < 0.0001), higher overweight preoccupation (*p* < 0.0001), and more frequently evaluating their weight as too high (*p* < 0.0001). HCEB was related to greater general internalisation of sociocultural ideals, greater internalisation of athletic ideals, and higher perceived pressures and information.

HCPAB was related to greater desire to lose weight (*p* < 0.0001) and higher physical activity (*p* < 0.0001, Table 4). Adolescents with higher HCPAB demonstrated lower appearance evaluation (*p* = 0.001), lower body satisfaction (*p* = 0.001), greater preoccupation with being overweight (*p* < 0.0001), and more frequently reported that their weight was too high (*p* < 0.0001). HCPAB was associated with higher internalisation of sociocultural ideals about appearance.

We tested the relationships between HCEB and HCPAB. Correlation analysis demonstrated a direct association between HCEB and HCPA, with a correlation coefficient of 0.409, *p* < 0.01.

Finally, we used multiple logistic regression analysis to predict the odds of HCEB and HCPAB from the study variables (Table 5 and Table 6). Female gender, HCPAB, overweight preoccupation, and body weight evaluation as too high (self-classified weight) significantly increased the odds of HCEB.

More frequent physical activity and HCEB were significantly associated with HCPAB, as was self-classified weight. Greater acceptance of general sociocultural ideals predicted lower HCEB, whereas the pressures subscale predicted higher HCEB.

## 4. Discussions

The present study aimed to assess the prevalence of HCSB and HCPAB in a sample of adolescents and to test associations with physical activity, internalisation of sociocultural attitudes towards appearance, and body image concerns. We expected that health-compromising eating and physical activity behaviours would be more prevalent among girls and adolescents with a higher BMI. The results of the study showed a relatively high prevalence of HCEB with a significantly higher prevalence in girls and participants with a higher BMI. Our results are in line with other studies showing a higher prevalence of HCEB in girls and adolescents with a higher BMI [22,24,25].

Our study demonstrated that the prevalence of adolescent HCPAB was higher than HCEB. Exercise addiction-related aspects, such as exercising for longer than two hours and participating in more than one training session, were most common in this sample. This finding is not surprising, as the overuse of exercise and compulsive exercising is a common purging strategy used to compensate for caloric intake [16]. Other HCPA behaviours were less popular, yet are worthy of concern, because they are associated with false physical activity beliefs and cause real damage to health (overuse of exercise, wearing impermeable clothes, and not drinking fluids during and after a workout). Contrary to our expectations, the prevalence of HCPAB was similar in boys and girls, yet the prevalence of HCPAB was higher in adolescents with a higher BMI. Pich et al. [31] showed that weight self-regulation in females attempted to combine diet and exercise, while boys relied almost exclusively on exercise. Studies also show that when attempting to meet the perceived high social standards of beauty, men develop a “drive for muscularity” and women develop a “drive for thinness” and higher physical activity is observed in boys who want to increase their weight and in girls who want to decrease body weight [33]. Dysfunctional exercising and HCPAB might therefore be equally prevalent in both genders.

In this study, we expected that higher physical activity would be a protective factor associated with the lower prevalence of HCEB. The present study demonstrated that adolescents without HCEB had higher physical activity and physical activity was not related to higher HCEB. The associations between physical activity and HCPAB were the opposite, however. Higher physical activity was associated with higher HCPAB and sufficiently physically active adolescents were more than three times more likely to use HCPAB. These findings might be explained by the fact that some exercising adolescents have higher body image concerns [46]. Data on the associations between body image concerns and physical activity is inconsistent. Some studies claim that exercise helps to improve body image in adolescent boys [47,48], however, other studies show that physical activity is not related to body image satisfaction [33,49]. The problem might also be explained by the lack of physical literacy education in schools and sports clubs. Chen et al. [50] demonstrated that although a physically active context in physical education helps students to make sense of fitness knowledge, a vigorous context shifts their attention away from cognitive learning towards physical participation. Physical education in Lithuania is mainly focused on engaging in physical activity, but not in learning about it. Martin et al. [51] demonstrated that adolescents’ physical activity is associated with higher weight controllability beliefs. It was speculated that body dissatisfaction together with weight controllability beliefs might contribute to disordered eating. This might also be the case for HCPAB, however, further studies should test these assumptions. We expected that adolescents in this study who engaged in HCEB would also demonstrate a higher prevalence of HCPAB. This assumption was confirmed, as HCPEB was the predictor of HCPAB and the opposite. Other studies also show relationships between dysfunctional eating and dysfunctional exercising [52].

Finally, we expected that higher internalisation of sociocultural attitudes towards appearance and body image concerns would be associated with HCEB and HCPAB, controlling by gender and BMI. As expected, this assumption was confirmed and this study clearly showed significantly higher body image concerns and internalisation of socially accepted beauty standards in the group with HCEB. Controlling by gender and BMI, however, we found that the variables of female gender, overweight preoccupation, and a tendency to evaluate own body weight as high or too high were the strongest predictors for HCEB. These findings are in line with those of other studies using adolescent samples [12,14,22,24,28] showing that gender, overweight preoccupation, and body weight dissatisfaction is associated with HCEB.

The associations of body image concerns, internalisation of sociocultural standards, and HCPAB were in the same direction, however, we found no significant relationship between appearance orientation and HCPAB. Controlling by gender and BMI, we found that evaluating body weight as high, internalised pressures to lose body weight, and higher physical activity were the strongest predictors for HCPAB. As the present study is one of the first studies to address this question, our understanding of these findings is limited. However, the fact that physical activity is a strong predictor of HCPAB is concerning and needs further examination. We found that adolescents with HCEB demonstrated lower self-esteem. This finding is in line with other studies [28]. This was not the case for the HCPAB, however. This might be explained by the fact that physical activity is associated with increased self-concept and self-worth in children and adolescents [53], and body image is the key element for self-esteem [54,55]. Further studies should test these findings. The strongest limitation of this study is the cross-sectional nature of the research. The cross-sectional nature of the study precluded an ability to establish temporal precedence between variables. Despite this limitation, this study provides useful insights for health promotion and education. The main findings of our study are in line with the conclusions of Fan and Jin [26], in that irrespective of current weight, adolescents who perceive themselves as overweight have a stronger intention to lose weight, but do not develop better eating and exercise habits. Health education in this domain is therefore crucial. Physical activity and exercise are the basis of an adolescent’s healthy lifestyle. However, this study adds to the knowledge that it is not enough to just promote physical activity in health education programs tackling healthy lifestyle, weight control, and body image concerns in adolescents. It is important to educate adolescents on how to use physical activity and exercise for weight control, helping them to avoid the false beliefs and scientifically disproven behaviours in the weight control and physical activity-related domain.

As the present study is one of the first studies to explore HCPAB in adolescents, a comparison with other results from other research is limited. As discussed earlier, the associations between physical activity and body image are unclear, and therefore studies should not only address this question, but also explore the associations between health literacy in the physical activity domain, weight control, and body image. Further studies should explore the prevalence of HPAB in other representative samples and compare results internationally. It is also important to explore the phenomena in students, recreational fitness exercisers, and amateur exercisers of different kinds of sports. The associations of HCPAB and HCEB with physical activity, body image concerns, exercise addiction, and other variables are important for future research on adolescents. These results are important for optimization of health promotion and education programs.

## 5. Conclusions

HCEB were more prevalent among girls, while there were no gender differences observed in HPAB. Adolescents with HCEB demonstrated lower self-esteem, poorer body image, and higher internalization of sociocultural attitudes towards an ideal appearance. Similar results were observed for HCPAB with the exception of self-esteem and appearance orientation. Controlling by gender and BMI, we found that overweight preoccupation, a tendency to evaluate own body weight as too high, and HCPAB were the strongest predictors for HCEB. Evaluating body weight as too high, internalised pressures to conform to beauty ideals, HCEB, and higher physical activity were the strongest predictors for HCPAB. HCPAB is an important variable associated with adolescents’ body image, physical activity, and weight control. These results have important implications for health promotion and education programs tackling adolescents’ healthy lifestyle, weight control, and body image concerns.

## Figures and Tables

**Table 1:** Descriptive characteristics of the sample.

Characteristics		Boys (*n* = 299)	Girls (*n* = 437)	*p*
Age, years, m ± SD		17.24 ± 0.60	17.24 ± 0.59	0.657
Body weight, %	Underweight	7.4	23.3	<0.0001
	Normal weight	81.6	72.8	
	Overweight	11.0	3.9	
Physical activity, %	Inactive	9.1	15.7	<0.0001
	Insufficiently active	39.3	48.3	
	Active	51.6	36.0	
Disordered eating ≥10 scores, %		14.4	34.2	<0.0001
Self-esteem, m ± SD		29.95 ± 4.72	28.95 ± 4.48	0.885
Difference between desired and estimated body mass, m ± SD		4.69 ± 10.59	−2.95 ± 6.00	<0.0001
MBSRQ, m ± SD	Appearance evaluation	3.20 ± 0.51	3.25 ± 0.47	0.393
	Appearance orientation	3.24 ± 0.45	3.61 ± 0.54	<0.0001
	Body areas satisfaction	3.57 ± 0.70	3.38 ± 0.68	<0.0001
	Overweight preoccupation	1.99 ± 0.78	2.58 ± 0.94	<0.0001
	Self-classified weight	2.82 ± 0.66	3.05 ± 0.62	<0.0001
SATAQ-3, m ± SD	General internalization	2.40 ± 0.89	2.89 ± 0.92	<0.0001
	Sport internalization	2.89 ± 0.92	2.77 ± 0.84	0.056
	Pressures	2.08 ± 0.72	2.43 ± 0.86	<0.0001
	Information	2.57 ± 0.71	2.80 ± 0.66	<0.0001

n = number, m = mean, SD = standard deviation, *p* = probability level, MBSRQ = the Multidimensional Body and Self Regulations Questionnaire-AS, SATAQ-3 = the Sociocultural Attitudes towards Appearance Questionnaire-3.

**Table 2:** Comparison of the health compromising eating and physical activity behaviours between boys and girls (%).

Variables	Boys (*n* = 299)	Girls (*n* = 437)	*p*
Health compromising eating behaviours			
Skip meals	20.1	52.2	<0.0001
Fast	10.0	29.7	<0.0001
Increase cigarettes smoked	16.4	14.2	0.413
Low calorie diet (≤800 kcal)	9.7	30.0	<0.0001
Use diuretics	5.7	7.1	0.447
One product or liquids diet	9.0	16.2	0.005
Use of diet pills	5.7	9.4	0.068
Vomit after meal	3.3	7.1	0.029
Use of laxatives	5.7	7.3	0.382
Health compromising physical activity behaviours			
Exercising longer than 2 h	40.5	43.7	0.383
Participating in more than one training session in series	33.4	29.3	0.231
Exercising several times per day	15.4	11.4	0.119
Wearing impermeable warm clothes for sweating	17.7	16.5	0.657
Exercise at the highest possible intensity	17.7	20.6	0.334
Avoiding drinking any fluids during and after workouts	23.7	22.9	0.786

n = number, % = percentage, *p* = probability level.

**Table 3:** Associations between adolescents’ health compromising eating behaviours and study variables.

Variables	Without HCEB, m ± SD	With HCEB, m ± SD	Mean Difference	Cohen’s d	*p*
Body mass index	20.4 ± 2.3	21.2 ± 2.7	−0.8	−0.3	<0.0001
Body weight discrepancy	3.1 ± 9.0	−2.3 ± 8.1	−5.4	0.7	<0.0001
Self-esteem	29.4 ± 4.8	28.5 ± 4.4	0.1	0.2	0.015
Physical activity, times/week	3.5 ± 1.8	3.2 ± 1.7	0.3	0.2	0.01
MBSRQ appearance evaluation	3.3 ± 0.5	3.2 ± 0.5	0.1	0.3	<0.0001
MBSRQ appearance orientation	3.4 ± 0.5	3.6 ± 0.6	−0.2	−0.4	<0.0001
MBSRQ body areas satisfaction	3.6 ± 0.6	3.3 ± 0.7	0.3	0.5	<0.0001
MBSRQ overweight preoccupation	1.8 ± 0.7	2.8 ± 0.9	−1.0	−1.3	<0.0001
MBSRQ self-classified weight	2.7 ± 0.6	3.2 ± 0.6	−0.5	−0.8	<0.0001
SATAQ-3 general internalization	2.5 ± 0.9	2.9 ± 0.9	−0.4	−0.5	<0.0001
SATAQ-3 athlete	2.7 ± 0.9	2.9 ± 0.9	−0.2	−0.2	<0.0001
SATAQ-3 pressures	2.0 ± 0.7	2.5 ± 0.9	−0.5	−0.6	<0.0001
SATAQ-3 information	2.6 ± 0.7	2.8 ± 0.7	−0.2	−0.4	<0.0001

m = mean, SD = standard deviation, *p* = probability level, MBSRQ = the Multidimensional Body and Self Regulations Questionnaire, SATAQ-3 = the Sociocultural Attitudes towards Appearance Questionnaire-3.

**Table 4:** Associations between adolescents’ health compromising physical activity behaviours and study variables.

Variables	Without HCPAB, m ± SD	With HCPAB, m ± SD	Mean Difference	Cohen’s d	*p*
Body mass index	20.3 ± 2.4	21.2 ± 2.6	−0.9	−0.3	<0.0001
Body weight discrepancy	1.5 ± 8.4	−0.8 ± 10.2	2.3	0.2	<0.0001
Physical activity, times/week	3.1 ± 1.7	3.6 ± 1.7	−0.5	−0.3	<0.0001
Self-esteem	29.0 ± 4.5	29.0 ± 4.7	0.0	0.0	0.958
MBSRQ appearance evaluation	3.3 ± 0.5	3.2 ± 0.5	0.1	0.2	0.001
MBSRQ appearance orientation	3.4 ± 0.5	3.5 ± 0.6	0.1	−0.07	0.362
MBSRQ body Areas satisfaction	3.5 ± 0.7	3.4 ± 0.7	0.1	0.2	0.001
MBSRQ overweight preoccupation	2.0 ± 0.8	2.6 ± 0.9	−0.6	−0.6	<0.0001
MBSRQ self-classified weight	2.8 ± 0.6	3.1 ± 0.6	−0.3	−0.5	<0.0001
SATAQ-3 general internalization	2.6 ± 0.9	2.8 ± 0.9	−0.2	−0.2	0.012
SATAQ-3 sport	2.7 ± 0.9	2.9 ± 0.9	−0.2	−0.3	<0.0001
SATAQ-3 pressures	2.1 ± 0.8	2.5 ± 0.8	−0.4	−0.5	<0.0001
SATAQ-3 information	2.6 ± 0.7	2.8 ± 0.7	−0.2	−0.2	0.001

m = mean, SD = standard deviation, *p* = probability level, BMI = body mass index, MBSRQ = the Multidimensional Body and Self Regulations Questionnaire, SATAQ-3 = the Sociocultural Attitudes towards Appearance Questionnaire-3.

**Table 5:** Multiple regression analysis predicting health compromising eating behaviours from study variables.

Predictors		OR	95% CI	*p*
Gender	boys	1.00		
	girls	1.88	1.10–3.18	0.02
Body mass index	normal	1.00		
	underweight	1.80	0.95–3.41	0.074
	overweight	0.59	0.23–1.52	0.274
Body weight discrepancy		0.99	0.95–1.04	0.672
Health compromising physical activity behaviour		1.19	1.10–1.28	<0.0001
Self-esteem		0.99	0.95–1.04	0.753
MBSRQ appearance evaluation		0.65	0.37–1.14	0.132
MBSRQ appearance orientation		1.19	0.78–2.13	0.330
MBSRQ body Areas satisfaction		0.83	0.57–1.19	0.307
MBSRQ overweight preoccupation		3.43	2.52–4.66	<0.0001
MBSRQ self-classified weight		2.40	1.57–3.68	<0.0001
SATAQ-3 general internalization		0.88	0.63–1.23	0.444
SATAQ-3 sport		0.93	0.69–1.24	0.599
SATAQ-3 pressures		1.15	0.83–1.61	0.406
SATAQ-3 information		1.15	0.79–1.68	0.469

OR = odds ratio, 95% CI = 95% confidence level, *p* = probability level, MBSRQ = the Multidimensional Body and Self Regulations Questionnaire, SATAQ-3 = the Sociocultural Attitudes towards Appearance Questionnaire-3.

**Table 6:** Multiple regression analysis predicting health compromising physical activity behaviours from study variables.

Predictors		OR	95% CI	*p*
Gender	boys	1.00		
	girls	0.74	0.46–1.18	0.206
Body mass index	normal	1.00		
	underweight	0.90	0.51–1.61	0.732
	overweight	0.93	0.41–2.12	0.854
Body weight discrepancy		1.00	0.98–1.02	0.952
Physical activity	0–1 days/week	1.00		
	2–3 days/week	1.73	1.03–2.93	0.04
	≥4 days/week	3.02	1.76–5.17	<0.0001
Health compromising eating behaviour		1.22	1.11–1.32	<0.0001
Self-esteem		1.02	0.98–1.06	0.339
MBSRQ appearance evaluation		0.79	0.48–1.28	0.334
MBSRQ appearance orientation		1.02	0.66–1.58	0.919
MBSRQ body Areas satisfaction		1.00	0.72–1.37	0.979
MBSRQ overweight preoccupation		1.32	0.99–1.75	0.058
MBSRQ self-classified weight		1.43	0.99–2.06	0.055
SATAQ-3 general internalization		0.70	0.52–0.94	0.018
SATAQ-3 sport		1.25	0.96–1.61	0.097
SATAQ-3 pressures		1.51	1.12–2.03	0.007
SATAQ-3 information		1.00	0.71–1.40	0.978

OR = odds ratio, 95% CI = 95% confidence level, *p* = probability level, MBSRQ = the Multidimensional Body and Self Regulations Questionnaire, SATAQ-3 = the Sociocultural Attitudes towards Appearance Questionnaire-3.

## References

[1] Poitras V.J., Gray C.E., Borghese M.M., Carson V., Chaput J.P., Janssen I., Katzmarzyk P.T., Pate R.R., Connor Gorber S., Kho M.E. (2016). Systematic review of the relationships between objectively measured physical activity and health indicators in school—Aged children and youth. Appl. Physiol. Nutr. Metab..

[2] WHO (2016). Growing Up Unequal: Gender and Socioeconomic Differences in Young People’s Health and Well-Being.

[3] Venckūnas T., Emeljanovas A., Mieziene B., Volbekiene V. (2017). Secular trends in physical fitness and body size in Lithuanian children and adolescents between 1992 and 2012. J. Epidemiol. Community Health.

[4] Smetanina N., Albaviciute E., Babinska V., Karinauskiene L., Albertsson-Wikland K., Petrauskiene A., Verkauskiene R. (2015). Prevalence of overweight/obesity in relation to dietary habits and lifestyle among 7-17 years old children and adolescents in Lithuania. BMC Public Health.

[5] Jankauskiene R., Baceviciene M. (2019). Body image concerns and body weight overestimation do not promote healthy behavior: Evidence from adolescents in Lithuania. Int. J. Environ. Res. Public Health.

[6] McLean S.A., Paxton S.J., Wertheim E.H., Masters J. (2015). Photoshopping the selfie: Self photo editing and photo investment are associated with body dissatisfaction in adolescent girls. Int. J. Eat. Disord..

[7] Anez E., Fornieles-Deu A., Fauquet-Ars J., López-Guimerà G., Puntí-Vidal J., Sánchez-Carracedo D. (2018). Body Image Dissatisfaction, physical activity and screen- time in Spanish adolescents. J. Health Psychol..

[8] Spencer R.A., Rehman L., Kirk S.F. (2015). Understanding gender norms, nutrition, and physical activity in adolescent girls: A scoping review. Int. J. Behav. Nutr. Phys. Act..

[9] Shaban L.H., Vaccaro J.A., Sukhram S.D., Huffman F.G. (2016). Perceived body image, eating behavior, and sedentary activities and body mass index categories in Kuwaiti female adolescents. Int. J. Pediatr..

[10] Gillison F.B., Standage M., Skevington S.M. (2006). Relationships among adolescents’ weight perceptions, exercise goals, exercise motivation, quality of life and leisure-time exercise behaviour: A self-determination theory approach. J. Health Educ. Res..

[11] Duchesne A., Dion J., Lalande D., Bégin C., Émond C., Lalande G., McDuff P. (2017). Body dissatisfaction and psychological distress in adolescents: Is self-esteem a mediator?. J. Health Psychol..

[12] Symons C., Polman R., Moor M., Borkoles E., Eime R., Harvey J., Craike M., Banting L., Payne W. (2013). The relationship between body image, physical activity, perceived health, and behavioural regulation among Year 7 and Year 11 girls from metropolitan and rural Australia. Ann. Leis. Res..

[13] Gatti E., Ionio C., Traficante D., Confalonieri E. (2014). “I Like My Body; Therefore, I Like Myself”: How Body Image Influences Self-Esteem—A Cross-Sectional Study on Italian Adolescents. Eur. J. Psychol..

[14] Lee J., Lee Y. (2016). The association of body image distortion with weight control behaviors, diet behaviors, physical activity, sadness, and suicidal ideation among Korean high school students: A cross-sectional study. BMC Public Health.

[15] Cruz-Zaez S., Pascual A., Salaberria K., Etxebarria I., Echeburúa E. (2015). Risky eating behaviors and beliefs among adolescent girls. J. Health Psychol..

[16] Voelker D.K., Reel J.J., Greenleaf C. (2015). Weight status and body image perceptions in adolescents: Current perspectives. Adolesc. Health Med. Ther..

[17] Craike M., Young J.A., Symons C.M., Pain M.D., Harvey J.T., Eime R.M., Payne W.R. (2016). Trends in body image of adolescent females in metropolitan and non-metropolitan regions: A longitudinal study. BMC Public Health.

[18] De Mar Bibiloni M., Pich J., Pons A., Tur J.A. (2013). Body image and eating patterns among adolescents. BMC Public Health.

[19] Gouveia M.J., Frontini R., Canavarro M.C., Moreira H. (2014). Quality of life and psychological functioning in pediatric obesity: The role of body image dissatisfaction between girls and boys of different ages. Qual. Life Res..

[20] Bucchianeri M.M., Arikian A.J., Hannan P.J., Eisenberg M.E., Neumark-Sztainer D. (2013). Body dissatisfaction from adolescence to young adulthood: Findings from a 10-year longitudinal study. Body Image.

[21] Marks D.F. (2015). Homeostatic theory of obesity. Health Psychol. Open.

[22] Neumark-Sztainer D., Wall M., Story M., Standish A.R. (2012). Dieting and Unhealthy Weight Control Behaviors During Adolescence: Associations With 10-Year Changes in Body Mass Index. J. Adolesc. Health.

[23] Neumark-Sztainer D., Story M., Dixon L.B., Murray D.M. (1998). Adolescents engaging in unhealthy weight control behaviors: Are they at risk for other health-compromising behaviors?. Am. J. Public Health.

[24] Stephen E.M., Rose J.S., Kenney L., Rosselli-Navarra F., Striegel Weissman R. (2014). Prevalence and correlates of unhealthy weight control behaviors: Findings from the national longitudinal study of adolescent health. J. Eat. Disord..

[25] Roy M., Gauvin L. (2013). Associations between different forms of body dissatisfaction and the use of weight-related behaviors among a representative population-based sample of adolescents. Eat. Weight Disord..

[26] Fan M., Jin Y. (2015). The Effects of Weight Perception on Adolescents’ Weight-Loss Intentions and Behaviors: Evidence from the Youth Risk Behavior Surveillance Survey. Int. J. Environ. Res. Public Health.

[27] Hsu Y., Liou T., Liou Y.M., Chien L.Y. (2016). Measurements and profiles of body weight misperceptions among Taiwanese teenagers: A national survey. Asia Pac. J. Clin. Nutr..

[28] Texeira M.D., Pereira A.T., Marques M.V., Saraiva J.M., de Macedo A.F. (2016). Eating behaviors, body image, perfectionism, and self-esteem in a sample of Portuguese girls. Rev. Bras. Psiquiatr..

[29] Lampard A.M., Maclehose R.F., Eisenberg M.E., Larson N.I., Davison K.K., Neumark-Sztainer D. (2015). Adolescents who engage exclusively in healthy weight control behaviors: Who are they?. Int. J. Behav. Nutr. Phys. Act..

[30] Stigler M.H., Arora M., Dhavan P., Shrivastav R., Reddy K.S., Perry C.L. (2011). Weight-related concerns and weight-control behaviors among overweight adolescents in Delhi, India: A cross-sectional study. Int. J. Behav. Nutr. Phys. Act..

[31] Pich J., del Mar Bibiloni M., Pons A., Tur J.A. (2015). Weight self-regulation process in adolescence: The relationship between control weight attitudes, behaviors, and body weight status. Front. Nutr..

[32] Bergier J., Niznijowskaja E., Bergier B., Acs P., Salonna F., Junger J. (2017). Differences in physical activity, nutritional behaviours, and body silhouette concern among boys and girls from selected European countries. Hum. Mov..

[33] Zach S., Zeev A., Dunsky A., Goldbourt U., Shimony T., Goldsmith R., Netz Y. (2013). Perceived body size versus healthy body size and physical activity among adolescents—Results of a national survey. Eur. J. Sport Sci..

[34] Dumitru D.C., Dumitru T., Maher A.J. (2018). A systematic review of exercise addiction: Examining gender differences. JPES.

[35] Hausenblas H.A., Schreiber K., Smoliga J.M. (2017). Addiction to exercise. BMJ.

[36] Cunningham H.E., Pearman S., Brewerton T.D. (2016). Conceptualizing primary and secondary pathological exercise using available measures of excessive exercise. Int. J. Eat. Disord..

[37] Paradis K.F., Cooke L.M., Martin L.J., Hall C.R. (2013). Too much of a good thing? Examining the relationship between passion for exercise and exercise dependence. Psychol. Sport Exerc..

[38] Longmuir P.E., Tremblay M.S. (2016). Top 10 research questions related to physical literacy. Res. Q. Exerc. Sport.

[39] Cole T.J., Lobstein T. (2012). Extended international (IOTF) body mass index cut-offs for thinness, overweight and obesity. Pediatr. Obes..

[40] Olfert M.D., Barr M.L., Charlier C.M., Famodu O.A., Zhou W., Mathews A.E., Byrd-Bredbenner C., Colby S.E. (2018). Self-reported vs. measured height, weight, and BMI in young adults. Int. J. Environ. Res. Public Health.

[41] Cash T.F. (2008). Body Image Assessments: Manuals and Questionnaires. http://www.body-images.com.

[42] Miskinyte A., Bagdonas A. (2010). Jaunų suaugusiųjų požiūrio į savo kūną sąsajos su demografiniais rodikliais (Associations between body image and demographic variables in young adults). Psichologija.

[43] Rosenberg M. (1965). Society and the Adolescent Self-Image.

[44] Thompson J.K., Van den Berg P., Roehrig M., Guarda A.S., Henberg L.J. (2004). The sociocultural attitudes towards appearance scale -3 (SATAQ-3): Development and validation. Int. J. Eat. Disord..

[45] Jankauskiene R., Mieziene B., Balciuniene V. (2016). Validation of the Lithuanian version of the Sociocultural Attitudes Towards Appearance Questionnaire-3. Soc. Behav. Person..

[46] Dyremyhr A.E., Diaz E., Meland E. (2014). How Adolescent Subjective Health and Satisfaction with Weight and Body Shape Are Related to Participation in Sports. J. Environ. Public Health.

[47] Campbell A., Hausenblas H. (2009). Effects of Exerscise Interventions on Body Image. J. Health Psychol..

[48] Gomez-Baya D., Mendoza R., Gaspar de Matos M., Tomico A. (2017). Sport participation, body satisfaction and depressive symptoms in adolescence: A moderated-mediation analysis of gender differences. Eur. J. Dev. Psychol..

[49] Laus M.F., Costa T.M., Almeida S.S. (2013). Body image dissatisfaction and aesthetic exercise in adolescents: Are they related?. Estudos Psicologia.

[50] Chen S., Chen A., Sun H., Zhu X. (2013). Physical activity and fitness knowledge learning in physical education: Seeking a common ground. Eur. Phys. Educ. Rev..

[51] Martin S.B., Rhea D.J., Greenleaf C.A., Judd D.E., Chambliss H.O. (2011). Weight control beliefs, body shape attitudes, and physical activity among adolescents. J. Sch. Health.

[52] Muller A., Boeber S., Sochtig S., Te Wildt B., de Zwaan M. (2015). Risk for exercise dependence, eating disorder pathology, alcohol use disorder and addictive behaviors among clients of fitness centers. J. Behav. Addict.

[53] Liu M., Wu L., Ming Q. (2015). How Does Physical Activity Intervention Improve Self-Esteem and Self-Concept in Children and Adolescents? Evidence from a Meta-Analysis. PLoS ONE.

[54] Badau D., Badau A. (2018). Identifying the Incidence of Exercise Dependence Attitudes, Levels of Body Perception, and Preferences for Use of Fitness Technology Monitoring. Int. J. Environ. Res. Public Health.

[55] Kololo H., Guszkowska M., Mazur J., Dzielska A. (2012). Self-efficacy, self-esteem and body image as psychological determinants of 15-year-old adolescents’ physical activity levels. Hum. Mov..

